# Potential Applications of Next-Generation Probiotics in the Development of Functional Food Products

**DOI:** 10.3390/molecules31213977

**Published:** 2026-11-04

**Authors:** Aleksandra Szydłowska, Barbara Sionek, Anna Łepecka

**Affiliations:** 1 Faculty of Health Sciences, Warsaw University of Life Sciences (WULS), Nowoursynowska St. 159C, 02-776 Warsaw, Poland; 2 Department of Meat and Fat Technology, Prof. Wacław Dąbrowski Institute of Agricultural and Food Biotechnology—State Research Institute, 02-532 Warsaw, Poland; anna.lepecka@ibprs.pl

**Keywords:** Next-Generation Probiotics (NGPs), functional food product development, *Akkermansia muciniphila*, *Faecalibacterium prausnitzii*, *Anaerobutyricum hallii*, *Prevotella copri*, *Clostridium butyricum*, *Bacteroides fragilis*, *Roseburia* spp.

## Abstract

Next-generation probiotics (NGPs), isolated from the human gut microbiome, represent a breakthrough direction in the development of precision functional foods and targeted biotherapeutics. This review article presents the current state of knowledge regarding the classification, mechanisms of action, and technological and regulatory challenges associated with the application of NGPs in functional food design. Representative commensal species (*Akkermansia muciniphila*, *Faecalibacterium prausnitzii*, *Anaerobutyricum hallii*, *Prevotella copri*, *Clostridium butyricum* and *Bacteroides fragilis*, *Roseburia* spp.) were analyzed to evaluate their capacity to maintain intestinal epithelial integrity, their efficiency in short-chain fatty acid (SCFA) synthesis, and their modulation of host immune responses. Their commercial use remains limited because most NGP strains are strict anaerobes that readily lose viability when exposed to oxygen, processing stress, or digestive fluids. Regulatory frameworks classify NGPs as live biotherapeutic products (LBPs) or Novel Foods, requiring rigorous safety evaluation and clinical trials. The effective implementation of NGP formulations in functional food production remains strictly dependent on the harmonization of global legislative criteria and advancements in the bioengineering of protective delivery systems, capable of maintaining cellular integrity and ensuring the biological activity of strains at the point of consumption of the final product.

## 1. Introduction

Diet is one of the most important factors shaping the intestinal microbiota because the quantity and quality of nutrients supplied influence the living conditions and activity of individual microorganisms [1]. Interest in health and the role of nutrition in maintaining it is constantly growing, which translates into the growing importance of functional foods [2]. The European project Functional Food Science in Europe proposed a definition of functional food, according to which “a food can be regarded as ‘functional’ if it is satisfactorily demonstrated to beneficially affect one or more target functions in the body, beyond adequate nutritional effects, in a way that is relevant to either an improved state of health and well-being and/or a reduction in the risk of disease. Functional foods must remain foods, and they must demonstrate their effects in amounts that can normally be expected to be consumed in the diet: they are not pills or capsules, but part of a normal dietary pattern” [3].

One important group of ingredients used in functional foods is probiotics. The International Scientific Association for Probiotics and Prebiotics (ISAPP) has clarified the term “probiotic” as “live microorganisms that, when administered in adequate amounts, confer a health benefit on the host” [4]. This definition primarily encompasses well-characterized strains belonging to the genera *Lactobacillus* (currently using a new nomenclature), *Bifidobacterium*, *Saccharomyces*, and certain *Bacillus* species, whose beneficial effects on health have been confirmed in scientific studies [4,5]. This action primarily involves modulating and maintaining gastrointestinal homeostasis [6].

In response to the specific needs of humans related to their microbiome, the concept of tailored probiotics has emerged. Their composition is selected based on the characteristics of the microbiome for a specific individual [7]. This allows us to avoid the mistakes associated with a universal probiotic, which would be health-effective for all consumers [6]. With the development of this concept, the idea of microbial strains with high health-promoting potential emerged, but these are not traditional probiotics [7]. A group of next-generation probiotics (NGPs) has been identified, which includes microorganisms naturally occurring in the human microbiota, but their biological effects are more focused on maintaining proper homeostasis of the intestinal microbiota [8], strengthening the integrity of the intestinal barrier [9], modulating the immune response [10], or influencing specific metabolic pathways [11]. Thus, the human gut microbiota has become a valuable but unknown source of potentially beneficial probiotic microorganisms [6]. In addition to gut microorganisms, NGPs can also be found in other isolation sources, particularly in the oral cavity, the female urogenital tract, on the skin, and in the nasopharynx. Environmental sources are also important [12].

Distinguishing NGPs from traditional probiotics has become possible as a result of the development of modern bioinformatics methods and next-generation sequencing (NGS), which have allowed for a much more accurate characterization of the human microbiota [13]. Of particular interest to scientists are commensal bacteria from the human microbiome, capable of producing short-chain fatty acids (SCFAs), which play an important role in regulating intestinal barrier function and the immune response. Therefore, NGPs constitute a promising group of microorganisms with beneficial health effects and may be used as adjuncts in the treatment of disorders associated with dysbiosis [1].

This review aims to synthesize the current state of knowledge regarding NGPs, focusing specifically on their definition and classification, mechanisms of action, key microbial strains, therapeutic benefits, legal regulations, and current functional food applications within this category.

## 2. Next-Generation Probiotics (NGPs)

### 2.1. Definition and Classification

To date, ISAPP has not formulated an official definition of NGPs. The term NGPs is used to refer to microorganisms identified from microbiome studies that exhibit health-promoting properties, but its scope remains a subject of scientific debate [14]. Definitely, NGPs correspond to the standard definition of probiotics but include microorganisms that have not yet been used as health-promoting agents [7]. O’Toole et al., 2017 [7] operationally refer to these organisms as “next-generation probiotics”, highlighting their potential development as novel microbial therapeutics and the overlap between NGPs and live biotherapeutic products. Compared to traditional probiotics, NGPs do not have a long history of use. NGPs are most often isolated from the human gastrointestinal tract, which should guarantee adaptation to the difficult intestinal environment and high bacterial counts [15]. Moreover, from a phylogenetic point of view, they are very distant from lactic acid bacteria (LAB) [1].

Al-Fakhrany & Elekhnawy, 2024 [6] proposed the following definition of NGPs: “living microbes identified on the basis of comparative microbiome investigations which confer health advantages to their host when taken to suitable extents”. This term appears to be expanded to include the process of identifying NGPs. Martin & Langella, 2019 [16] proposed a definition encompassing “live microorganisms identified on the basis of comparative microbiota analyses that, when administered in adequate amounts, confer a health benefit on the host”. In turn, a new publication by Kern et al. [15] defines NGPs as “live microorganisms identified through advanced comparative analysis of the gut microbiome, using next-generation sequencing and bioinformatics to isolate bacteria with defined health benefits”. The concept of NGPs should be distinguished from the term “live biotherapeutic products” (LBP), which refers to a category of biological products defined by the U.S. Food and Drug Administration (FDA). According to the FDA definition, LBP is a biological product that contains live organisms, such as bacteria; is applicable to the prevention, treatment, or cure of a disease or condition of human beings; and is not a vaccine [17]. Although the categories of NGPs and LBP may partially overlap, they are not equivalent. NGPs primarily describes a group of microbial candidates considered for their health-promoting potential, whereas classification as LBP depends on the product’s characteristics, its intended use, and the applicable regulatory framework.

In summary, NGPs constitute a new generation of microorganisms with potential therapeutic applications that go beyond the traditional definition of probiotics.

**Figure 1:**
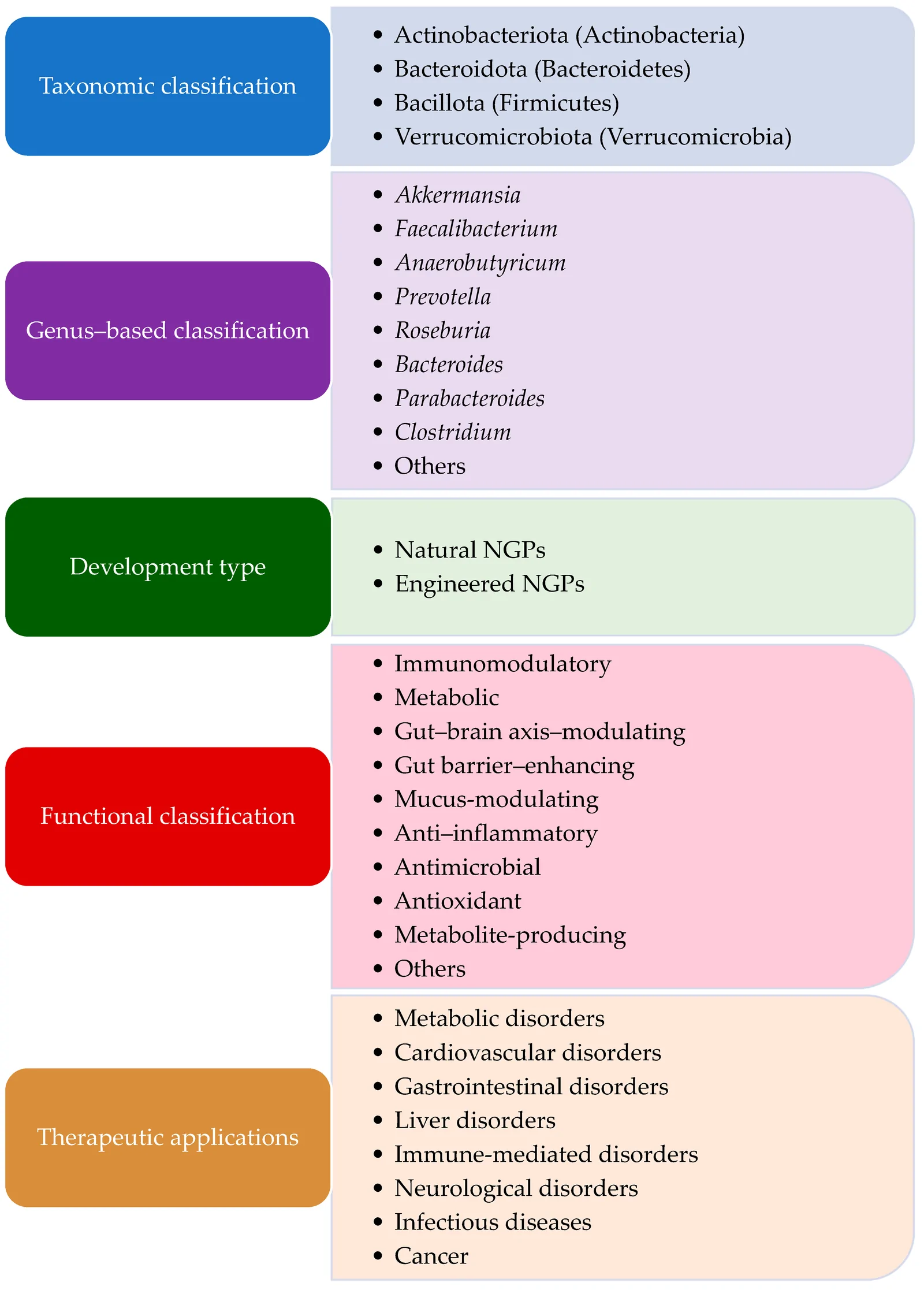
Classification of the next–generation probiotics. The blue color indicates taxonomic classification. The purple color indicates genus–based classification. The green color indicates development type. The red color indicates functional classification. The orange color indicates therapeutic applications. Prepared by the authors based on De Filippis et al., 2022 [1]; Kern et al., 2026 [15], Jan et al., 2024 [18].

As with definitions, there is no official classification of NGPs. Various divisions of NGPs are proposed in Figure 1.

The Figure presents a multi-criteria characterization of NGPs. These microorganisms are organized according to five criteria: taxonomic affiliation, type, method of production, functional properties, and potential therapeutic applications [15].

NGPs encompass potential probiotic microorganisms from diverse taxonomic groups, primarily gut-associated bacteria belonging to the phyla Actinobacteriota, Bacteroidota, Bacillota, and Verrucomicrobiota. Representative genera include Akkermansia, *Faecalibacterium, Anaerobutyricum, Prevotella, Roseburia, Bacteroides, Parabacteroides*, and *Clostridium* among others In terms of how NGPs are obtained, a distinction can be made between natural and engineered types. Natural NGPs are those isolated from the natural environment (human gut microbiota) whose beneficial properties stem from their inherent biological characteristics. Examples include bacteria from the genera *Akkermansia, Faecalibacterium, Roseburia,* and specific strains of *Clostridium butyricum*. Engineered NGPs, by contrast, are microorganisms whose properties have been intentionally altered through genetic engineering or synthetic biology. These modifications are designed to introduce new functions or enhance the activity of existing biological mechanisms. Examples include bacteria from the genera *Escherichia, Lactococcus*, and *Bacteroides* [15,18]. This classification demonstrates the diversity of NGPs and their potential in the prevention and treatment of various diseases. However, their beneficial effects and therapeutic potential are strain-dependent and require appropriate safety and efficacy evaluation [15].

### 2.2. Mechanisms of Action of NGPs

The mechanisms of action of NGPs are complex and involve interactions between microorganisms, the host, and the intestinal environment. It is estimated that the mechanisms of action of NGPs are more complex than those of traditional probiotics [18]. At the same time, it is emphasized that the mechanisms are species-dependent and even strain-dependent [15].

**Figure 2:**
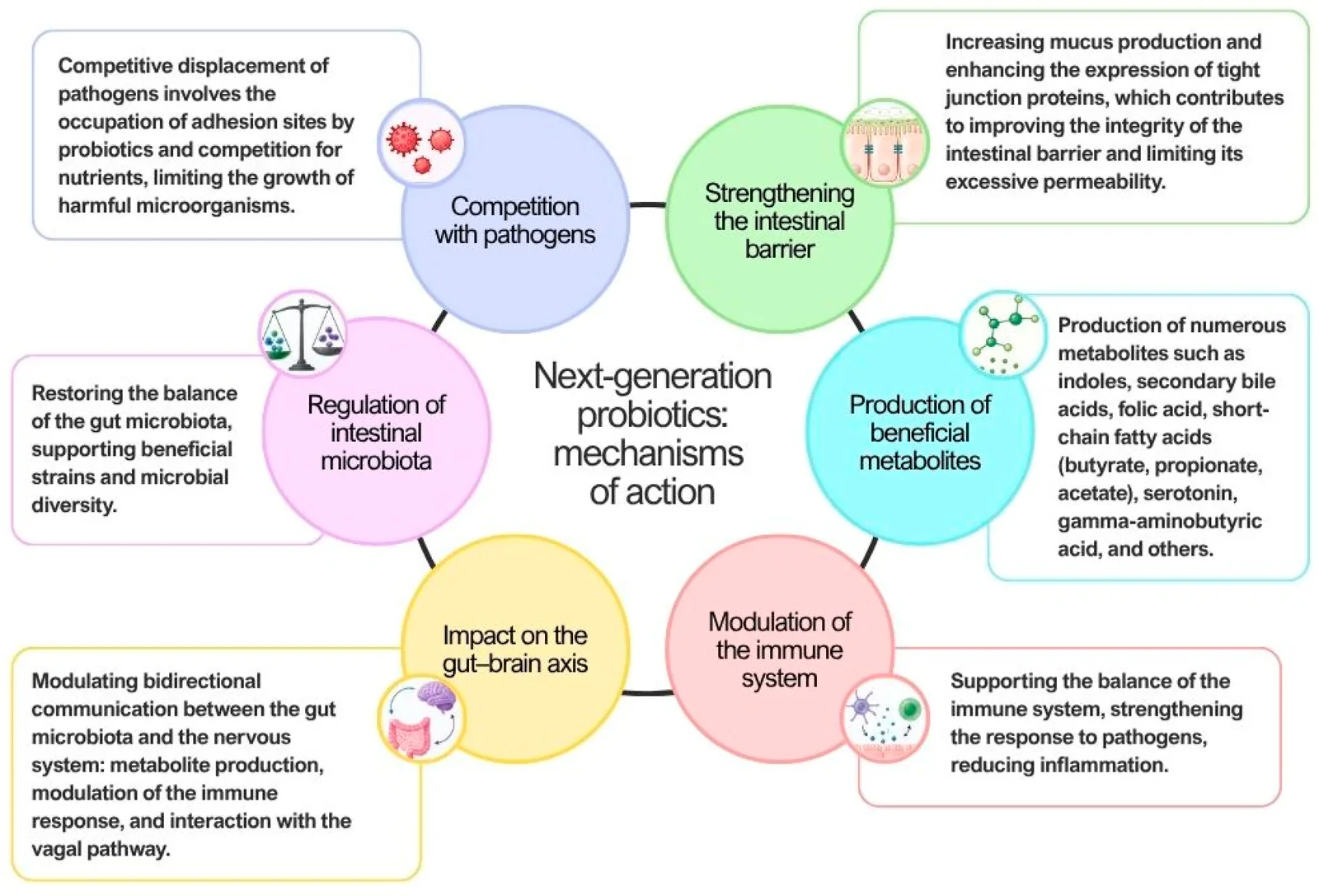
Mechanisms of action of next-generation probiotics related to strengthening the intestinal barrier (marked in green), production of beneficial metabolites (marked in blue), modulation of the immune system (marked in red), influence on the gut–brain axis (marked in yellow), regulation of microbiota (marked in pink), and competition with other microorganisms (marked in purple). Prepared by the authors based on Abouelela & Helmy, 2024 [13]; Al-Fakhrany & Elekhnawy, 2024 [6]. Created with the free version of Canva.

The most important mechanisms of action of NGPs include strengthening the intestinal barrier, production of beneficial metabolites, modulation of the immune system, influence on the gut–brain axis, regulation of the gut microbiota, and competition with pathogens (Figure 2).

Strengthening the intestinal barrier is a complex process that includes improving the integrity of the intestinal epithelium, maintaining homeostasis, and influencing the expression and organization of tight junction proteins. Mucus secretion is crucial for maintaining barrier integrity [19]. The proper functioning of individual components of the intestinal barrier contributes to limiting intestinal hyperpermeability and preventing the translocation of undesirable bacteria and their metabolites into host tissues [20]. Consequently, improving the functioning of individual components of the intestinal barrier plays a key role in maintaining homeostasis [21]. *Akkermansia muciniphila* plays a particular role in this function, promoting mucin production and strengthening tight junction proteins, thus strengthening the integrity of the intestinal barrier [9,11].

NGPs are widely known for producing metabolites with beneficial effects. The most important of these are SCFAs, particularly butyrate, propionate, and acetate, which are formed primarily through the fermentation of undigested carbohydrates. Their production depends strictly on the substrates available in the intestine [13]. Indoles, secondary bile acids, and folic acids, as well as trimethylamine-N-oxide and serotonin have also been characterized. These metabolites play a role in regulating host physiological phenotypes [8]. Some of them act as signaling molecules in metabolic processes [13]. Gamma-aminobutyric acid (GABA) is a neuroactive metabolite that acts as an inhibitory neurotransmitter in the nervous system and influences the functioning of the gut–brain axis [22]. Beneficial metabolites also include antimicrobial compounds, such as lactic acid, biologically active peptides, and bacteriocins, which can limit the growth of pathogens [13].

Another mechanism of action involves the influence of NGPs on the immune system. Bacteria are capable of modulating the immune response by stimulating the production of both anti-inflammatory and pro-inflammatory cytokines. Furthermore, it has been demonstrated that interactions between bacteria and immune cells can lead to the activation of specific signaling pathways [23]. Consequently, modulation of the immune response by NGPs may contribute to maintaining immune homeostasis within the gut [15].

As mentioned earlier, GABA acts as a mediator of the gut–brain axis and plays a significant role in brain function. Its levels in the brain are tightly regulated by the microbiota, which in turn has a significant impact on the modulation of mental health. Homocarnosine is the dominant GABA-containing brain peptide and is an important component of this system. Therefore, modulation of the gut–brain axis can be assumed to be one of the mechanisms supporting neuroplasticity and the neuroprotective effects of NGPs [22].

Maintaining the gut microbiota at an appropriate level is crucial for maintaining gastrointestinal homeostasis. NGPs are capable of modifying the environment by altering nutrient availability, metabolite production, and metabolic interactions with other microorganisms. This leads to the stability of the gut ecosystem, creating an interconnected mechanism for maintaining homeostasis [15].

NGPs compete with other microorganisms for nutrients and habitat in the gut, thereby inhibiting the growth of pathogens and simultaneously strengthening the intestinal barrier [13]. Limiting colonization by potentially harmful microorganisms helps maintain microbial balance in the gut [15].

In summary, the mechanisms of action of NGPs are multifaceted, complementary processes. The interplay of these mechanisms contributes to the beneficial effects of NGPs on host health.

### 2.3. NGPs Microorganism Candidates and Health Benefits

*Akkermansia muciniphila*, *Faecalibacterium prausnitzii*, *Anaerobutyricum hallii*, *Prevotella copri*, *Roseburia* spp., *Bacteroides fragilis*, *Parabacteroides goldsteinii*, and *Clostridium butyricum* are considered the most promising NGPs [1]. These bacteria are characterized below.

#### 2.3.1. *Akkermansia* (*A.*) *muciniphila*

*A. muciniphila* is an obligate anaerobic, non-motile, non-spore-forming Gram-negative bacterium that was first isolated from the feces of a healthy human by the Dutch microbiologist Muriel Derrien [24]. Taxonomically, *A. muciniphila* belongs to the phylum *Verrucomicrobiota* (formerly *Verrucomicrobia, Verrucomicrobaeota*) [25]. Its characteristic feature is its ability to utilize mucin as its primary source of carbon, nitrogen, and energy. The term “muciniphila” means “mucin-loving,” a group of highly glycosylated proteins, called glycoproteins, which in the human body constitute the main component of the mucus layer covering the mucous membranes of the gastrointestinal tract, urogenital, and respiratory tracts [11]. The ability to utilize mucin allows it to persist in the intestinal mucus layer even during periods of limited nutrient availability [25].

According to Cunningham et al., 2021 [12], *A. muciniphila* is one of the most promising NGPs, and despite its specific requirements for rich growth media and anaerobic conditions, its potential is very high. For this reason, it is often referred to as the “star probiotic” [11]. In the human gut microbiome, it constitutes 0.5 to 5% of the total number of bacteria [24]. The highest numbers of *A. muciniphila* are found in the cecum, with the number of these bacteria influenced by many factors, including age, diet and lifestyle, diseases, medications, and antibiotics [25]. Of crucial importance is the fact that overly low *A. muciniphila* counts are closely linked to the development of cardiovascular disease, obesity, diabetes, nonalcoholic fatty liver disease, and autism [10,25]. Low levels of *A. muciniphila* may lead to a lack of intestinal barrier homeostasis, which allows toxins to enter the host [6].

At the same time, changes in *A. muciniphila* counts may be considered a biomarker of metabolic diseases [25]. Therefore, *A. muciniphila* may be viewed as a potential therapeutic element in the prevention and treatment of obesity and type 2 diabetes [11,26]. This is confirmed by the studies by Suenaert et al., 2026 [27], where pasteurized *A. muciniphila* MucT improved intestinal barrier integrity in individuals with obesity and prediabetes. Furthermore, body weight and trunk fat tissue reduction were achieved, which may offer patients an opportunity to improve body composition parameters [27]. Preclinical research by Mount et al., 2026 [28], showed that the group of patients supplemented with pasteurized MucT had lower weight gain compared to the control group. In contrast, the study by Plovier et al., 2017 [29] demonstrated the efficacy of both live and pasteurized *A. muciniphila* bacterial cells, but pasteurized bacteria were more effective. This phenomenon is associated with the presence of a specific outer membrane protein, Amuc_1100, which is responsible for regulating inflammatory and metabolic processes and also strengthens the intestinal barrier, fulfilling a key postbiotic role [29]. A direct interaction of the Amuc_1100 protein with Toll-like receptor 2 (TLR2) has been demonstrated, which may contribute to strengthening tight junctions and increasing the integrity of the host intestinal barrier [1].

Despite many promising research results, there is still insufficient data to clearly determine the optimal dose of *A. muciniphila* and assess the safety of its long-term use. In particular, there are no descriptions of the negative effects of *A. muciniphila* use [11,25,28].

#### 2.3.2. *Faecalibacterium* (*F.*) *prausnitzii*

One of the most abundant and well-studied bacteria in the human gastrointestinal tract is *F. prausnitzii* [6]. In healthy humans, it constitutes approximately 5% of the gut microbiome. It is an obligately anaerobic, non-spore-forming, non-motile rod, a Gram-variable bacterium with irregular or negative staining. This staining variability depends on the strain and method used. It belongs to the Bacillota (*Firmicutes*) phylum, class Clostridia [30]. It is capable of fermenting starch, oligofructose, inulin, fructose, and glucose. Therefore, a diet rich in fiber promotes its growth [6].

Its unique feature is the presence of glucose fermentation products, particularly SCFAs, such as formic acid, butyrate, small amounts of D-lactic acid, as well as medium-chain fatty acids and salicylic acid. These products demonstrate the significant health-promoting potential of this bacterium [6]. Butyrate is particularly important, playing a crucial role in intestinal function as an important energy source for colonocytes. The high metabolic activity of *F. prausnitzii* positively affects colonocytes, thus maintaining immune balance and carbohydrate and lipid metabolism and regulating the host’s energy processes [30]. Butyrate synthesis by *F. prausnitzii* occurs via butyryl-CoA:acetate-CoA transferase (BCoAT), which catalyzes the conversion of butyryl-CoA to butyrate using acetate as a substrate. Although butyrate can also be synthesized by butyrate kinase in the gut microbiota, this pathway is not specific to *F. prausnitzii* [31].

The usefulness of determining *F. prausnitzii* abundance is very broad. For example, the number of these bacteria can be a good marker of adolescent obesity [32]. Furthermore, studies by Zhang et al., 2014 [33], confirmed the anti-inflammatory effects of *F. prausnitzii*, with these effects being exerted not only by the bacterial cells but also by the culture supernatant. An increase in plasma anti-Th17 cytokines (IL-10 and IL-12) and a decrease in IL-17 levels were noted, which alleviated inflammatory changes in the rat colon. The authors of the study suggest that metabolites and other compounds secreted during *F. prausnitzii* growth may be involved in inhibiting proinflammatory pathways and reducing the production of inflammatory mediators [33]. A meta-analysis by Cao et al., 2014 [34], demonstrated that the microbiota of patients with Inflammatory Bowel Disease (IBD) is characterized by low *F. prausnitzii* counts, and that dysbiosis plays a role in the etiology and pathogenesis of IBD. Additionally, it was found that patients with dysbiosis can effectively use probiotics to restore the balance of the gut microbiota, particularly butyric acid-producing microorganisms [34].

In summary, *F. prausnitzii* is one of the best-known representatives of the bacteria known as NGPs, primarily due to its potential importance in maintaining intestinal homeostasis.

#### 2.3.3. *Anaerobutyricum* (*A.*) *hallii*

In 2018, a taxonomic reclassification of previously described *Eubacterium* species was proposed. Due to changes in nomenclature, *Eubacterium hallii* was renamed *Anaerobutyricum hallii* [35]. These bacteria are non-spore-forming and obligately anaerobic, belonging to the Lachnospiraceae family [6]. *A. hallii* was originally isolated from human feces. Gram-positive *A. hallii* are a typical component of the human large intestine microbiota, accounting for approximately 3% of all microorganisms. They exhibit extensive colonization of the gastrointestinal tract [6]. The number of *Anaerobutyricum* spp. bacteria is influenced by a number of factors, including age, physiological state, human disease, and diet [36].

Comprehensive genomic analysis of *A. hallii* demonstrated that these bacteria lack virulence factors, confirming some safety characteristics. The increased genomic plasticity of *A. hallii* primarily demonstrates their high ability to adapt to changing host environmental conditions [37]. The ability of *A. hallii* to utilize various carbon sources, e.g., sugars and organic acids, enables its growth and participation in fermentation and SCFAs production [36]. The production of propionate and butyrate is particularly important, depending on the available substrate [38].

The phenomenon of cross-feeding, which involves metabolic cooperation between bacteria, is extremely interesting. *A. hallii* can utilize lactate produced by other microorganisms (e.g., *Bifidobacterium adolescentis*) and convert it to butyrate. The mechanism of action involves the transfer of carbon from lactate via acetyl-coenzyme A to butyrate [39]. Lactate utilization offers a dual benefit, as lactate accumulation may be associated with intestinal dysfunction [36].

Studies in mice have shown that oral administration of *A. hallii* can improve insulin sensitivity and increase energy expenditure. Increasing butyrate concentration in the feces of mice modified bile acid metabolism and, consequently, improved gut microbiome function [40]. Furthermore, studies by Ramirez Garcia et al., 2023 [38], demonstrated the ability of *A. hallii* to modify the harmful effects of carcinogenic xenobiotics. The mechanism of action involves contributing to the transformation of the dietary carcinogen PhIP (2-amino-1-methyl-6-phenylimidazo [4,5-b]pyridine), leading to the formation of products with reduced cytotoxicity and mutagenicity [38].

Knowledge about *A. hallii* is very limited, and its mechanisms of action are still poorly described. Primarily, more isolates are needed for research, which is difficult because these bacteria are strictly anaerobic and require specific nutrients for growth [36].

#### 2.3.4. *Prevotella* (*P.*) *copri*

In humans, *P. copri* occurs in the intestines, vaginal area, and oral cavity. Morphologically, the bacterium is Gram-negative, strictly anaerobic, non-spore-forming, and incapable of producing spores [41,42]. *P. copri* is considered difficult to isolate and culture [43]. A characteristic feature of *P. copri* is its ability to utilize complex polysaccharides and convert dietary fiber into SCFAs, succinic acid, and other metabolites. Therefore, *P. copri* is found to be abundant in humans whose diet is high in fiber [43].

As mentioned earlier, the resulting SCFAs protect the mucosal barrier and reduce the risk of inflammation [41]. From the perspective of NGPs, it is an extremely interesting bacterium due to its dual nature. On the one hand, it plays a beneficial role in the intestinal ecosystem, exhibiting anti-inflammatory, antidiabetic, anti-allergic, and metabolic regulatory effects in the liver [42]. Oral administration of *P. copri* to mice increases glucagon-like peptide-1 (GLP-1) secretion and improves hyperglycemia, insulin resistance, and lipid abnormalities, alleviating pathological changes in the pancreas, liver, and colon [43]. Furthermore, it has been found that *P. copri* can influence the pool and proportions of bile acids, participating in their metabolism, thus modulating the intestinal environment and host metabolic processes. It has been noted that low *P. copri* counts after antibiotic therapy can result in a lower ratio of primary to secondary bile salts [41].

On the other hand, some studies have indicated that *P. copri* may exhibit pro-inflammatory potential. It should be emphasized, however, that these associations do not necessarily imply a direct causal link. The impact of *P. copri* on the host depends on the specific strain, its metabolic potential, the environmental context, and the composition of the gut microbiota [41].

In summary, *P. copri* possesses a diverse biological potential, and its effect on host health can be either beneficial or detrimental. The potential use of specific *P. copri* strains as ingredients in functional foods or as probiotic candidates requires a thorough assessment of their functional properties, safety, and efficacy through preclinical and clinical studies.

#### 2.3.5. *Roseburia* spp.

*Roseburia* spp. are Gram-positive, anaerobic, curved rods belonging to the family *Lachnospiraceae*, phylum formerly known as *Firmicutes*, class Clostridia. Some *Roseburia* spp. are motile and have flagella. *Roseburia* spp. were first isolated from feces and are considered difficult to culture and isolate [44]. The abundance of *Roseburia* spp. in the human microbiome ranges from 3 to 15% [6], of which R. intestinalis accounts for 0.9 to 5.0% of the total microbiota. The most popular species include *R. intestinalis*, *R. hominis*, *R. inulinivorans*, *R. faecis*, and *R. cecicola* [45].

Like other commensal intestinal bacteria, they are capable of producing SCFAs—particularly butyrate, which is formed during fiber fermentation. Studies indicate that R. intestinalis and *F. prausnitzii* are among the most important butyrate producers in the human intestine, and their importance results from both their high butyrogenic activity and relatively high abundance in the gut microbiota [44]. *Roseburia* spp. utilize various fiber components, such as xylans and β-mannans, which promote the production of SCFAs [46]. *Roseburia* spp. owe their ability to produce butyrate to butyryl-CoA:acetate CoA-transferase, an enzyme that catalyzes the final step of butyrate biosynthesis [44].

Research has observed that the number of *Roseburia* spp. is low in patients with colon cancer and inflammatory diseases. Therefore, *Roseburia* spp. are believed to play a significant role in reducing intestinal inflammation [47]. A review by Nie et al., 2021 [44], described that *R. intestinalis* is capable of producing beneficial metabolites, which influences homeostasis and thus inhibits inflammation in the intestines. Jiang et al., 2025 [48], demonstrated that R. intestinalis alleviates neuropathic pain in mice, potentially through the production of butyrate and the activation of free fatty acid receptor 3 (FFAR3/GPR41) signaling in vagal afferents, thereby modulating the gut–brain axis. However, this proposed mechanism should be interpreted with caution, as evidence for the involvement of FFAR3/GPR41 signaling in pain modulation remains preliminary and is derived from preclinical animal studies. Further research is needed to establish the relevance of this pathway in humans and to determine the potential of *R. intestinalis* as an adjunctive therapeutic strategy for neuropathic pain [48]. Other studies suggest that *R. intestinalis* may limit the development of Crohn’s disease pathogenesis by stimulating anti-inflammatory mechanisms. *R. intestinalis* was found to increase the number of Treg cells and the expression of anti-inflammatory cytokines TSLP, TGF-β, and IL-10 [49].

Abnormal numbers of *Roseburia* spp. are observed in metabolic disorders and diseases associated with intestinal dysbiosis, further indicating their potential importance for host health and providing broad potential as NGPs.

#### 2.3.6. *Bacteroides* (*B.*) *fragilis*

The anaerobic Gram-negative bacterium *B. fragilis* constitutes approximately 1% of the human gut microbiota [6]. *B. fragilis* is a commensal, pleomorphic, anaerobic, non-motile, non-spore-forming rod, often encapsulating. Like other NGPs, *Bacteroides* spp. produce SCFAs from indigestible glycans derived from the diet. *Bacteroides* spp. do not digest mucin unless it is the sole nutrient for growth. With limited fiber availability, metabolism may be shifted towards mucin degradation [50].

*B. fragilis* is a commensal bacterium of the gut microbiota, but also an opportunistic pathogen. It is believed that some strains of *B. fragilis* may produce a toxin with zinc-dependent metalloprotease activity, which can damage the intestinal barrier. Depending on the presence of the bft gene encoding the BFT toxin (*Bacteroides fragilis* toxin), *B. fragilis* is divided into two groups: non-enterotoxigenic *B. fragilis* and enterotoxigenic *B. fragilis* (ETBF) [1]. ETBF may contribute to the development of cancer. The infection can be asymptomatic, and the consequence is the development of colon tumors. Research has shown that the presence of *B. fragilis* in patients with colon cancer was relatively common, while the presence of the bft gene, indicating the enterotoxic nature of the strain, was observed in some patients [51].

Scientific studies have demonstrated the health-promoting effects of *B. fragilis*, including alleviating colitis, restoring normal intestinal microbiota after antibiotic therapy, and maintaining intestinal homeostasis [50]. These properties are attributed to polysaccharide A (PSA), membrane vesicles (OMVs), SCFAs, and other polysaccharides. PSA favorably influences the host immune response by activating the TLR2 pathway and promoting the production of anti-inflammatory interleukins [52]. Studies by He et al., 2023 [53], demonstrated the anti-inflammatory effects of *B. fragilis* ATCC 25285 by alleviating colitis and improving intestinal barrier function. The metabolic supernatant of *B. fragilis* was also found to possess anti-inflammatory properties.

As emphasized by De Filippis et al., 2022 [1], *B. fragilis* as a candidate for NGPs must be thoroughly tested for safety due to its dual nature.

#### 2.3.7. *Parabacteroides* (*P.*) *goldsteinii*

*P. goldsteinii*, along with *P. distasoni*, is one of the most studied species of the genus *Parabacteroides*. Previously, this species was classified as *Bacteroides goldsteinii* [54]. *P. goldsteinii* is a common commensal bacterium in the human gastrointestinal tract and was first isolated from the feces of healthy adults. These are obligately anaerobic, Gram-negative rods that produce acetate and succinate by metabolizing glucose. Their numbers are influenced by diet, pharmacological agents, and lifestyle [55]. Low or abnormal *P. goldsteinii* numbers are observed in metabolic disorders associated with obesity. Studies in mice have shown that stimulating the production of succinate and secondary bile acids stabilized intestinal permeability, reduced endotoxin levels, and decreased insulin resistance [54].

Lai et al., 2022 [56], discovered a link between the gut microbiota and lung axis. *P. goldsteinii* alleviates the course of chronic obstructive pulmonary disease (COPD) by inhibiting inflammation, restoring normal amino acid metabolism, and increasing the activity of cellular organelles, particularly mitochondria and ribosomes, in the large intestine. Furthermore, promising results were obtained with *P. goldsteinii* lipopolysaccharide, which also exhibits anti-inflammatory properties by blocking the activation of Toll-like receptor 4 (TLR4) [56].

In another study, Lai et al. [57] demonstrated the properties of *P. goldsteinii* MTS01, which modulated the composition of the gut microbiota and reduced the pathogenesis of *Helicobacter pylori*. A significant reduction in cholesterol levels and a reduction in gastric inflammation through antivirulence were demonstrated, weakening the harmful effects of VacA and CagA factors on gastric epithelial cells. The authors indicate that *P. goldsteinii* MTS01 has great potential as an NGP [57].

#### 2.3.8. *Clostridium* (*C.*) *butyricum*

Many species of the genus *Clostridium* are components of the gut microbiota and can positively influence intestinal homeostasis by interacting with epithelial cells, strengthening the integrity of the intestinal barrier, and producing SCFAs [18]. *C. butyricum* derives its name precisely from its ability to ferment indigestible carbohydrates and produce SCFAs, specifically, butyrate. It is a Gram-positive, anaerobic, spore-forming bacterium [6]. It is commonly found in the human colon, where it participates in the metabolic processes of the gut microbiota [7].

The beneficial effects of *C. butyricum* primarily include reducing the frequency of persistent diarrhea associated with intestinal diseases. Successful therapeutic outcomes have also been observed in patients with ulcerative colitis, a condition characterized by alterations in the gut microbiota [18]. Furthermore, *C. butyricum* has been shown to inhibit the proliferation of cancer cells in the gut [6]. Strains of this species are used in the treatment of *Clostridium difficile* and *Helicobacter pylori* infections [7]. *C. butyricum* is a compelling candidate as an NGP, primarily due to its capacity to produce significant amounts of butyrate and its multifaceted potential to influence the gut microbiota.

The presented bacteria belong to the group of NGPs, which, due to their biological properties and potential health-promoting effects, constitute a promising direction of research on intestinal microbiota.

## 3. Safety and Legal Regulations for the Use of NGPs in the USA, Europe, and Selected Asian Countries

The main sources of probiotics are the digestive tracts of humans and animals, fermented foods (mainly dairy), breast milk, and other sources, such as plant products [58,59]. In recent years, advances in genetic engineering technologies—particularly molecular methods, those based on sequencing of the 16S rRNA gene—have revolutionized knowledge of the gastrointestinal microbiome. The number of identified environmental and clinical bacterial species has significantly increased [60]. New genetic typing tools have made it possible to distinguish a new group of probiotic microorganisms in the human microbiome classified as NGPs [7]. Microorganisms classified as NGPs have enormous promising therapeutic potential, specific to a selected group of individuals and conditions [61]. Despite the dynamic development of genetic engineering technologies and the passage of more than two decades, legal regulations concerning probiotics have not changed proportionately and remain within the existing framework created on the basis of definitions and standards established years ago. This is most likely due to safety concerns. There is an awareness of the risks associated with the introduction of new microorganisms into food, like horizontal gene transfer, virulence factors, and persistent colonization.

NGPs, as a new group, cannot be treated like traditional probiotics, e.g., those with a long history of safe use and meeting U.S. Generally Recognized As Safe (GRAS) status. In addition, NGPs have never been used in food so far. Reports from available studies and meta-analyses about the positive health effects of NGPs are insufficient for the authorities to accept them in accordance with the applicable standards. Without obtaining the appropriate status of a “health claim” for the entire population and carrying out a lengthy and cost-intensive evaluation, they cannot be included in the European Qualified Presumption of Safety (QPS) list either. The QPS list contains microorganisms intentionally added to food and feed. The QPS list, first established in 2007, has been continuously revised and updated. The QPS process was developed to provide a harmonized safety assessment approach in accordance with the European Food Safety Authority (EFSA) established under EU law in 2002 in order to provide the scientific basis for laws and regulations to protect European consumers from food-related risks [62]. A generic qualification for all QPS bacterial taxonomic units applies in relation to the absence of acquired genes conferring resistance to clinically relevant antimicrobials [63,64]. NGPs are living microorganisms, with a status referred to as a “medical claim” intended to be used in the defined target population [65]. Therefore, they are qualified as LBPs and are considered biological medical products. The U.S. FDA guidelines for developing LBPs were published in 2012 [17]. If NGPs were to be registered as a “medical claim”, i.e., effective in populations with a specific disease, then they should be subjected to the rules of registration as LBPs and they should be considered as medical products [65]. According to the FDA: “An LBP, for the purposes of this guidance document, is a biological product that: (1) contains live organisms, such as bacteria; (2) is applicable to the prevention, treatment, or cure of a disease or condition of human beings; and (3) is not a vaccine”. Since NGPs are defined as LBPs, i.e., drugs, and their main purpose of consumption is considered therapeutic, according to regulations in the U.S., an Investigational New Drug Application (IND) is required before the clinical trials phase. This IND for an LBP focuses on the chemistry, manufacturing, and control (CMC) information that should be submitted [66]. In the next step, NGPs must undergo phase I, II, and III of costly and long-term clinical trials, including efficacy, relative benefit, and side-effect analysis in large groups of the target population [67].

If NGPs will be treated like traditional probiotics present in food products, their evaluation should be subjected to a “health claim” appropriate for functional foods [65]. In 2022, the U.S. FDA approved the first microbiota product—Rebyota, a fecal transplant, indicated for the prevention of recurrence of *Clostridioides difficile* (*C. difficile*) infection in individuals 18 years of age and older, following antibiotic treatment for recurrent *C. difficile* infection [68]. REBYOTA is prepared from human fecal material collected from pre-screened and qualified donors and tested for prespecified pathogens. Each 150 mL dose of REBYOTA contains between 1 × 10^8^ and 5 × 10^10^ colony-forming units per mL of fecal microbes, including >1 × 10^5^ CFU/mL of Bacteroides. It works by facilitating the restoration of the gut flora to prevent further episodes of *Clostridioides difficile* infection [69,70]. In 2023, the FDA approved the first oral medicine—VOWST, indicated to prevent the recurrence of *C. difficile* infection in individuals 18 years of age and older following antibacterial treatment for recurrent *C. difficile*. VOWST is a bacterial spore suspension in capsules for oral administration. Each capsule contains 1 × 10^6^ to 3 × 10^7^ spore colony-forming units. VOWST is prepared from stool donations obtained from qualified healthy donors based on scientific risk assessment [71].

In Europe, food-related health claims are evaluated by EFSA [72]. According to the European Commission Recommendation 2015/2283, a high level of protection of human health and of consumers’ interests should be ensured. Emerging technologies in food production processes may have an impact on food and thereby on food safety. Therefore, this Regulation should further specify that a food should be considered a novel food where it results from a production process not used for food production within the Union before 15 May 1997, which gives rise to significant changes in the composition or structure of a food affecting its nutritional value, metabolism or level of undesirable substances [73]. EFSA guidelines were last updated in 2018. This guidance document refers to the data needed for the assessment of sources of nutrients proposed for use in the manufacture of foods. It describes the scientific data required to allow an evaluation of the safety of the source within the established framework for risk assessment and the bioavailability of the nutrient from this source, but it does not overlap with NGPs or LBPs [74]. In 2022, under EU Regulation 2022/68 on extracellular vesicles, pasteurized *A. muciniphila* was authorized as a novel food, for use at up to 3.4 × 10^10^ cells/day in food supplements and in food for special medical purposes, for the adult population, excluding pregnant and lactating women [75]. In 2024, the EFSA Panel on Biological Hazards reassessed the suitability of *A. muciniphila* for qualified presumption of safety (QPS) status. According to BIOHAZ, *A. muciniphila* was still not recommended for QPS status due to safety concerns [8]. Recently (23 February 2026), based on new scientific evidence, the EU approved an extension of its use to the general population aged 12–18, with differentiated maximum intake levels by age group (Regulation (EU) 2026/391) [76]. In this complicated and time-consuming way, mainly considering safety, the pasteurized strain of *A. muciniphila* became the first NGPs to be approved as a novel food [76].

The system for regulating medicines in Europe is unique. It is based on a closely coordinated regulatory network of national competent authorities in the Member States of the European Economic Area, working together with the European Medicines Agency (EMA) and the European Commission. In 2019, the European Pharmacopoeia (Eu Ph) set requirements for LBP with the adoption of quality standards. They defined LBPs as medicinal products containing living microorganisms such as bacteria or yeasts, which have a positive influence on the health and physiology of the host. Requirements of Eu Ph include a full morphological, biochemical, serological, and molecular characterization of the strains used (since the therapeutic characteristics are strain-specific), as well as ensuring the absence of antimicrobial resistance or any other virulence factors in LBPs. [77]. The European Medicines Agency regulates medicines for human use in the EU and plays a key role in the approval of medicines. The EMA is guided by the requirements for applications for centralized marketing authorisation for medical products valid in all EU Member States. So far, the EMA has not dealt with live microorganisms, including LBP. There is no ready-to-use protocol for the evaluation and registration of LBP [65]. However, scientific documents from the European Medicines Agency state that available guidelines on biological human medicines help applicants prepare marketing authorisation applications [78]. The Pharmabiotic Research Institute Center (PRI), as the European Microbiome Regulatory Science Expertise center, was created in 2010 to support the development and registration of therapeutic and diagnostic products emerging from microbiome science. According to the PRI any other medicinal products, microbiome-based medicinal products must comply with the pharmaceutical standards for quality, safety and efficacy, and will require a medicinal product marketing authorization to reach market [79,80]. In 2025, the European Commission launched a legislative initiative to establish the “EU Biotech Act” [81]. The Biotech Act will propose a series of measures to create an enabling environment to accelerate the transition of biotech products from laboratory to factory and to the market while maintaining the highest safety standards for the protection of the population and the environment. Regulation provides the basis for the assurance of a high level of protection of human health and consumers’ interests in relation to food, taking into particular account the diversity in the supply of food, including traditional products, whilst ensuring the effective functioning of the internal market. It establishes common principles and responsibilities, the means to provide a strong scientific basis, efficient organizational arrangements and procedures to underpin decision-making in matters of food and feed safety. It covers medicinal products within the meaning of Council Directive 2001/83/EC. The Biotech Act hopefully will enable the creation of an environment for convenient and safe marketing authorization of a medicinal product in the EU: from laboratory to factory and to a medicinal product [82,83].

Asia is the largest and fastest growing region in the probiotics market; however, probiotic regulations vary from country to country [84]. The functional food concept and first regulations were developed in 1991 in Japan as ‘Food for Specified Health Uses’ (FOSHU). Currently, Japanese regulations distinguish three categories of foods with health claims: FOSHU includes food with probiotics, Foods with Nutrient Function claim (FNFC) includes food with probiotics, and Food with Function Claim (FFC) includes vitamins and minerals, not probiotics. Consumer Affairs Agency is responsible for the approval of food as FOSHU. The FFC launched in 2015 based on notification system with the responsibility of the operator (Japan’s Consumer Affairs Agency does not evaluate the safety and effectiveness of function claims) is more flexible and enabled manufactures to introduce novel functional food products [80,85]. In China, probiotic regulations were initially issued in 1995 in the “Food Sanitation Law”, replaced by the Food Safety Law in 2009 and revised in 2015 [80]. In Aug 2026 China’s State Administration for Market Regulation and the National Technical Committee for Standardization of Special Foods issued the draft national standard ‘Probiotic Ingredients for Health Foods’. It covers the following: 1. Clear definitions of probiotics. 2. Strain-specific identification. 3. Strict production control. 4. Defined quality and safety limits. 5. Post-market safety monitoring. 6. Enhanced labeling requirements [86]. The new standards introduced with the Japanese FFC and Chinese ‘Probiotic Ingredients for Health Foods’, while promoting closer integration between the application of scientific achievements and industry, do not create separate regulations for NGPs.

A major challenge for NGPs is the lack of uniform global standards for the assessment and approval of new microorganisms. Each country has a regulatory and legislative body. The registration requirements vary significantly from one region of the world to another, and are not harmonized across all jurisdictions. However, the fundamental difference is discrimination between a “medical claim” dedicated to a drug (intended for use by a specific sick group of patients), and a “health claim” designed for a food or a food supplement (for use by the healthy population) [65,66]. These rules pose a significant global challenge for companies looking to develop NGPs for use by human subjects. Irrespective of different regulatory rules across the world, high quality, high performance, and constant product reproducibility are essential. This poses an economic challenge for companies looking to introduce and market foods containing NGPs or other NGP-containing products [17].

## 4. Development of Functional Food Products Containing NGP

A contemporary approach to functional foods is shifting from traditional fermented products toward precision and personalized dietary interventions [87,88]. This sector, historically dominated by dairy products containing conventional LAB of the genera *Lactobacillus* and *Bifidobacterium*, is currently facing a critical demand for deep technological innovation [9,88]. This necessity is driven both by the physiological limitations of traditional dairy-based carriers—such as lactose intolerance, which affects approximately 75% of the global population, and milk protein allergies—and by a growing consumer demand for precision nutrition and foods with targeted therapeutic efficacy [87,89].

To address these challenges, researchers are focusing on integrating next-generation probiotics (NGPs) into innovative food matrices. NGPs are highly specialized commensal microbes of human gut origin, such as *Akkermansia muciniphila*, *Faecalibacterium prausnitzii*, and *Bacteroides* species (e.g., *Bacteroides fragilis*). Incorporating these unique microflora into newly designed functional food products offers revolutionary opportunities for the prevention and management of chronic lifestyle-related diseases, including metabolic, immunological, dermatological, and gastrointestinal disorders [9,89].

Nonetheless, the industrial-scale application of NGPs in the food sector is hindered by strict technological and regulatory barriers [6,90]. As obligate anaerobes, these microorganisms are extremely sensitive to oxygen exposure and thermal stress during processing and storage, which drastically compromises their cell viability and stability [4]. Additionally, a key prerequisite for their biological efficacy is ensuring high survival rates during transit through the highly acidic gastric environment and in the presence of bile salts. This is essential for delivering the bacteria to the colon in therapeutic doses, typically targeted at a range of 6–7 log CFU per 1 g of product [87,91].

Current solutions in the field of food engineering are based on the implementation of innovative solutions, such as microencapsulation or Layer-by-Layer (LbL) assembly, which allows for limiting the adverse impact of environmental conditions on sensitive bacterial cells [6,9,91]. Concurrently, breakthrough research on non-viable alternatives, such as pasteurized NGP strains, their postbiotics, and extracellular vesicles (EVs) offers a way to obtain highly stable functional products. These alternatives completely eliminate the technological risks associated with oxygen and thermal sensitivity while preserving immunogenic and metabolic efficacy [91,92].

The successful implementation of probiotics (with a particular focus on NGPs) into advanced functional food matrices and biotherapeutic formulations is strictly contingent upon overcoming both physiological and technological barriers. For the administered microorganisms to exert a desired health-promoting effect on the host, a critical prerequisite is maintaining their stability and a minimum therapeutic dose conventionally defined in the literature at 10^6^–10^7^ CFU/g (or CFU/mL) at the time of consumption, as well as ensuring their survival during passage through the human gastrointestinal tract [90,93].

The primary challenge to microbial viability following oral administration stems from the harsh conditions encountered across different sections of the human gastrointestinal tract, namely the extreme acidity of gastric juice, as well as the action of digestive enzymes and bile salts. The low pH of gastric fluid destroys unprotected bacterial cell walls and cell membranes, leading to the destabilization of their membrane potential [87]. Resistance to these stress conditions is closely correlated with the specific genus and species of the microorganism. Bacteria of the genus *Bifidobacterium* exhibit low stability at pH 2.0–3.0, whereas *Lactobacillus* species are characterized by higher acid tolerance [6]. Upon reaching the duodenum, bacterial cells are exposed to pancreatic enzymes and bile salts, which disrupt the lipid bilayers of bacterial cell membranes, ultimately inducing cell lysis [6,94]. Microenvironmental factors during intestinal transit also play a critical role. Cellular viability and adhesive capacity are further constrained by fluctuations in osmotic pressure, the presence of specific ions, nutrient depletion in the intestinal lumen, and shear forces generated by intestinal peristalsis [6]. It should be emphasized that ensuring the stability of food products fortified with next-generation probiotics (NGPs) also requires protecting these sensitive microorganisms from extreme stress conditions during manufacturing and commercial distribution [95,96].

A critical issue is the extreme sensitivity of these microorganisms to oxygen and oxidative stress. Unlike traditional LAB, most key candidates for next-generation probiotics (NGPs) (e.g., *Faecalibacterium prausnitzii, Bacteroides fragilis*) are obligate intestinal anaerobes. They are extremely sensitive to atmospheric oxygen and undergo rapid cell death in oxic environments [97]. Even *Akkermansia muciniphila*, despite possessing mechanisms that allow it to tolerate very low oxygen levels (microaerophilia), is classified as a strict anaerobe and exhibits high oxygen sensitivity [98,99]. This characteristic poses major hurdles for their large-scale industrial production, requiring expensive culture media and the rigorous maintenance of anoxic conditions [91].

Thermal and mechanical stress during manufacturing processes also adversely affect the quality of functional food products fortified with NGPs. Classical dehydration techniques employed in the food industry carry a high risk of destroying biomass. Spray drying involves atomising the bacterial suspension and subjecting it to hot air (often reaching temperatures up to 220 °C), generating severe thermal, dehydrative, and mechanical stresses that disrupt cellular integrity. Although freeze-drying is considered a gentler alternative, freezing and ice crystal formation similarly cause the physical disruption of bacterial membranes, necessitating the use of costly cryoprotectants [90,100,101].

Furthermore, a major technological challenge lies in incorporating NGPs into plant-based food matrices (e.g., functional beverages and foods stored at room temperature), where bacteria are subjected to the cumulative effects of dissolved oxygen, ambient temperature fluctuations, and low pH due to natural organic acids. These factors induce oxidative, osmotic, and dehydrative stresses in bacterial cells, leading to membrane disruption and a drastic decline in bacterial counts (log CFU/g or mL). In contrast, traditional dairy products—typically distributed under refrigerated conditions (approx. 4–5 °C) and possessing higher buffering capacity—provide a substantially more stable environment that markedly limits probiotic biomass degradation [102,103].

The introduction of next-generation probiotics (NGPs), including obligate anaerobes of intestinal origin, into commercial food products and oral formulations necessitates overcoming critical technological and physiological barriers [100].

The technological efficacy and long-term storage viability of probiotic cultures are fundamentally governed by the structural and physicochemical properties of the selected food vehicle. Enclosing microorganisms within dehydrated, powder-based microcapsules provides a robust protective shield against environmental stressors and facilitates controlled intestinal delivery. Hence, the engineering and optimization of probiotic encapsulation systems necessitate a rigorous, multifaceted evaluation encompassing cellular integrity, functional activity, safety profiles, and release precision [87,104].

To overcome the technological limitations of conventional dehydration methods (discussed in this Section), novel biopolymer-based delivery systems (such as responsive hydrogels, LbL multilayer coatings, or nanofibers) are being developed to ensure high encapsulation efficiency and targeted intestinal release [100,105].

Table 1 presents examples of functional food vehicles and delivery systems incorporating next-generation probiotics (NGPs).

In conclusion, based on the data presented in Table 1, overcoming technological barriers and the harsh conditions of the gastrointestinal tract is achievable through the application of suitable food matrices and targeted bio-encapsulation processes in the production of NGP-containing functional foods. These strategies ensure high cell viability and preserve the biological activity of the specified strains.

## 5. Challenges and Future Directions

Beyond the survival hurdles addressed in Section 4, the successful commercialisation of NGPs in functional foods is constrained by food-matrix interactions, strain-level safety requirements, and complex regulatory frameworks. In specific food vehicles (e.g., fermented plant bases or high-polyphenol chocolate), constituents such as organic acids, osmotic fluctuations, or polyphenolic compounds can compromise long-term microbial viability during shelf-life storage.

Unlike traditional probiotics with Generally Recognized as Safe (GRAS) status, emerging NGPs are novel human gut commensals that require comprehensive strain-level biosafety validation prior to market entry. High-throughput Whole-Genome Sequencing (WGS) and comparative genomics must be routinely deployed to verify the absence of virulence factors, toxin-encoding sequences, and mobile elements conferring antimicrobial resistance. Regulatory standards remain fragmented globally: in the United States, strains with disease-targeted claims are classified as live biotherapeutic products (LBPs) requiring Investigational New Drug (IND) applications, whereas in the European Union, approval as Novel Foods under EFSA oversight restricts health-claim labeling. Establishing harmonized regulatory pathways in this aspect remains a huge challenge.

Future technological advancements should focus on next-generation delivery systems, particularly stimuli-responsive “smart” hydrogels tailored for targeted colonic release, as well as non-viable postbiotic alternatives (e.g., pasteurized cells and extracellular vesicles) to bypass oxygen sensitivity limitations altogether.

## 6. Conclusions

New generation probiotics (NGPs) exhibit great potential for use in the development of functional foods thanks to their targeted metabolic actions, which strengthen the gut barrier and exert immunomodulatory effects. However, commercializing these microorganisms remains challenging because of features such as high sensitivity to oxygen, processing stress, and passage through the digestive system. This issue requires the development of advanced protective systems such as hydrogels, nanocapsules or inanimate postbiotics, with the simultaneous establishment of harmonious legal requirements for novel food and LBPs. The combination of effective protection of strains with real regulatory solutions is an opportunity to introduce safe and effective functional products containing NGPs.

## Figures and Tables

**Table 1:** Examples of functional food matrix and delivery systems incorporating next-generation probiotics (NGPs).

Functional Food Matrix	NGP Strain	Delivery System	Health Benefits of Used Strains	Reference
Dark and medium-dark chocolate	*Akkermansia muciniphila* DSM 22959	Direct incorporation of free cells into the tempered cocoa mass (37 °C) or freeze-drying microencapsulation within a xanthan/gellan gum polymer matrix.	The hydrophobic cocoa butter lipid matrix isolates bacterial cells from oxygen exposure. Maintains cellular viability at 6 log CFU/g after 28 days of aerobic storage (54.5% cocoa) and over 60 days of anaerobic storage. Supports gut mucosal barrier integrity.	[104,106]
Synbiotic beverages	*Faecalibacterium prausnitzii*	Immobilization within alginate-starch hydrogel beads or LbL nanocoating with fructose/inulin, combined with co-culturing with Desulfovibrio piger to support anaerobic growth.	Protects cells from gastric acidity and oxygen toxicity. Stimulates the efficient production of butyrate and Microbial Anti-inflammatory Molecules, which inhibit the NF-κB signaling pathway and attenuate colonic inflammation (IBD).	[90,96,107]
Fermented dairy beverages	*Anaerobutyricum hallii* (formerly *Eubacterium hallii*)	Exploitation of trophic cross-feeding dynamics with infant *Bifidobacterium* species, which degrade human milk oligosaccharides into lactate and acetate.	*A. hallii* utilizes cross-feeding intermediates to synthesize butyrate, propionate, reuterin, and pseudovitamin B_12_, thereby improving host insulin sensitivity and energy homeostasis.	[6]
Functional beverages	*Clostridium butyricum*	Direct formulation of bacterial endospores into the food matrix or spore encapsulation within citrus peel pectin polysaccharides.	Endospore formation provides natural physiological resistance to thermal processing and gastric transit. Germinates in the colon to yield high levels of butyric acid, conferring anti-cancer activity, modulating the gut–brain axis, and alleviating colitis.	[108]

## Data Availability

The original contributions presented in this study are included in the article. Further inquiries can be directed to the corresponding authors.

## Abbreviations

The following abbreviations are used in this manuscript:

NGPs	Next-Generation Probiotics
SCFAs	Short-Chain Fatty Acids
LBPs	Live Biotherapeutic Products
FUFOSE	Functional Food Science in Europe
ISAPP	The International Scientific Association for Probiotics and Prebiotics
NGS	Next-Generation Sequencing
LBP	Live Biotherapeutic Product
FDA	Food And Drug Administration
TMAO	Trimethylamine-N-Oxide
GABA	Gamma-Aminobutyric Acid
CVD	Cardiovascular Disease
NAFLD	Nonalcoholic Fatty Liver Disease
TLR2	Toll-Like Receptor 2
BCoAT	Butyryl-Coa: Acetate-CoA Transferase
IBD	Inflammatory Bowel Disease
PhIP	2-amino-1-methyl-6-phenylimidazo [4,5-b]pyridine
NTBF	Non-Enterotoxigenic *B. fragilis*
ETBF	Enterotoxigenic *B. Fragilis*
PSA	Polysaccharide A
OMVs	Membrane Vesicles
COPD	Chronic Obstructive Pulmonary Disease
TLR4	Toll-Like Receptor 4
LBL	Layer-by-Layer
